# Are There “Carriers” of the B. Tetanus?

**Published:** 1918-01

**Authors:** 


***Are There " Carriers " of the B. Tetanus?*** A Report by Dr. Sylvio COLOMBINO, Surgeon of the Military Hospital of Monteortone (Italy), presented by M. LEGUEU to the *Société de Chirurgie de Paris* at its meeting on Dec. 20, 1916. Trans. and Abs. from the *Bulletins et Mémoires*, Dec. 26, 1916.

L. recalls that Berard and Lumière[1] in 1915 called attention to the fact that ragged wounds may retain tetanus spores in a latent state, temporarily isolated in a sheath of tissues or in fleshy granulations. In this manner he explained late tetanus. C. in the report here presented, however, maintains that not only the spores, but also the bacilli, may remain latent in this manner. He further believes that such organisms may be dangerous to others as well as to the man on whom they are found, thus constituting a danger of contagion.

C. first had his attention called to the problem by the fact that only 2 men out of 200 whose wounds were treated preventively by serum, developed tetanus. This led him to suppose that in many of the rest the tetanus organisms must have been present, especially

1. *Lyon Médical*, Oct. 1915, t. XII, n° 4, p. 404.

since the serum injections could not in any way act as a bactericide.

In several cases he proved the presence of the tetanus organism, although no symptoms of tetanus were observed.

Lest such organisms constitute a source of infection for other men, C. urges that a preventive treatment of serum be administered not only before operations upon those known to have the tetanus organism, but also before operations *upon other patients* as well.

C., however, presents no case in which such contamination is known to have taken place.

In the *discussion* following the report, M. Delbet stated that he believed that such transmission of the disease was a real danger. He cited a case in which a woman upon whom he had operated developed tetanus apparently without cause. It was later discovered that on the farm where she worked there was an epidemic of tetanus among the horses. He did not think, however, that the likelihood of such transmission was very great.

MM. Quénu and Broca stated that they had never used preventive injections of tetanus antitoxin for operations upon patients who were not suspected of being infected, and they saw no need of such precaution, provided that the usual prophylactic measures were taken, and the tetanus patients properly isolated.

Most of the speakers approved of preventive injections against tetanus administered as a matter of routine after injuries, but there appeared to be no definite grounds for supposing that injections were necessary before all operations as a safeguard against " contagion at a distance ".

